# Phylogenetic relationships of fluorescent pseudomonads deduced from the sequence analysis of 16S rRNA, *Pseudomonas*-specific and *rpo*D genes

**DOI:** 10.1007/s13205-016-0386-x

**Published:** 2016-02-23

**Authors:** Asmita Rajwar, Manvika Sahgal

**Affiliations:** Department of Microbiology, College of Basic Sciences and Humanities, Govind Ballabh Pant University of Agriculture and Technology, Pantnagar, US Nagar, Uttarakhand India

**Keywords:** *Pseudomonas*, 16S rDNA, Genus-specific gene, *rpo*D gene, Sequence analysis, Molecular taxonomy

## Abstract

Phylogenetic relationship of 22 FLPs was revealed on the basis of polymorphism in three genes namely 16S rDNA, *Pseudomonas*-specific and *rpo*D gene regions. The primers for 16S rDNA, *Pseudomonas*-specific region and *rpo*D gene region were amplifying a region of 1492, 990 and 760 bp, respectively, from all the isolates investigated. The RFLP analysis of the PCR products resulted in a classification of these fluorescent pseudomonads which was best answered by *rpo*D-based RFLP analysis. The 22 FLPs were placed in two major clusters and seven subclusters suggesting that these were genotypically heterogenous and might belong to several species within *Pseudomonas* sensu stricto. Sequence analysis of these three genes for three selected isolates AS5, AS7 and AS15 showed 16S rDNA and *Pseudomonas*-specific gene region phylogenies were generally similar, but *rpo*D gene phylogeny was somewhat different from these two genes. These results were also congruent with the results of RFLP of these three genes. *rpo*D provided comparable phylogenetic resolution to that of the 16S rRNA and *Pseudomonas*-specific genes at all taxonomic levels, except between closely related organisms (species and subspecies levels), for which it provided better resolution. This is particularly relevant in the context of a growing number of studies focusing on subspecies diversity, in which single-copy protein-encoding genes such as *rpo*D could complement and better justify the information provided by the 16S rRNA gene. Hence *rpo*D can be used further as an evolutionary chronometer for species-level identification.

## Introduction

Molecular microbial ecology commenced in 1990 with the direct amplification and sequencing of 16S rRNA genes from the environment (Giovannoni et al. 1990). This procedure revolutionized microbial ecology and permanently changed the way we study prokaryotes in the environment. rRNA genes could identify an organism by reconstructing its phylogeny, along with the possibility of storing sequences in databases, resulting in the rapid adoption of the 16S rRNA gene by microbiologists. However, none of the 16S rRNA-based molecular methods allows for an accurate representation of microbial communities. It is also created by the existence of multiple heterogeneous copies of the 16S rRNA gene within a genome (Crosby and Criddle 2003; Dahllof et al. 2000). It seems that the resolution of 16S rRNA-based analysis is low due to the small numbers of substitutions between compared 16S rRNA sequences. Some of the studies have identified organisms with identical 16S rRNA gene sequences that have significant sequence divergence in protein-encoding genes (Papke et al. 2003; Pernthaler and Pernthaler 2005). The use of a single-copy gene for community analysis is an important milestone in microbial ecology, as it could allow for the accurate measurement of diversity and phylogenetic relationships, avoiding a loss in phylogenetic resolution and biases in diversity measurements due to the presence of intragenomic heterogeneity (Case et al. 2006). To resolve the phylogenetic relationships of closely related organisms, it would be necessary to use other gene sequences which provide a higher resolution than that of 16S rRNA. A rapid and accurate system for the identification of *Pseudomonas* isolates is essential in order to determine or monitor their role in the environment. Yamamoto et al. (2000) proposed the analysis of protein-encoding genes (*gyr*B and *rpo*D) for the discrimination of *Pseudomonas* species. These genes evolve much faster than rRNAs and provide higher resolution than the analysis of 16S rRNA gene sequences. In addition to their utility in the identification of *Pseudomonas* strains, the discriminating power of *rpo*D gene sequences was also reported in previous studies on the taxonomy of the genus *Pseudomonas* (the *Pseudomonas* *stutzeri* genomovars and *Pseudomonas* *corrugata* groups) (Guasp et al. 2000; Cladera et al. 2004; Mulet et al. 2008).

In this study, a comparison between the 16S rRNA, *Pseudomonas*-specific and *rpo*D genes is performed in order to evaluate the use of an alternative gene as a marker for molecular microbial ecology. We finally address whether the *rpo*D gene fulfills the criteria required for a molecular marker in microbial ecology. Protein-encoding genes have been reported to evolve much faster than rRNAs (Ochman and Wilson 1987); thus a phylogenetic analysis using the *rpo*D sequences was expected to provide higher resolution than one using 16S rRNA sequences.

## Materials and methods

### Isolation of fluorescent pseudomonads and genomic DNA

In all, 22 pseudomonad isolates were recovered from soil samples; grasslands of Pithoragarh (latitude—29°35′N and longitude—80°15′N, altitude—1615 masl) and forests of Almora (latitude—29°37′N and longitude 79°40′E, altitude—1250 masl); central Himalayan region in Uttarakhand using King’s B and Gould’s medium (data not shown). All these isolates were screened for their antagonistic potential (Gould et al. 1984). The genomic DNA was extracted by a modified method of Bazzicalupo and Fani (1995).

### PCR amplification of target genes of 16S rDNA, *Pseudomonas*-specific gene region and *rpo*D gene region

Genomic DNA was amplified by using three different PCR primer sets—one set was of universal primers (Anzai et al. 2000), other one was of *Pseudomonas*-specific PCR (Ps-PCR) primers (Widmer et al. 1998) and third one was for housekeeping gene *rpo*D-specific primers (Mulet et al. 2009).

The universal primer set included forward primer GM3f (5′ AGAGTTTGATCMTGGC 3′) and reverse primer GM4r (5′ TACCTTGTTACGACTT 3′) for the amplification of 1492 bp region of the 16S rDNA gene. A 50 μl of reaction mixture included, 5 μl (5–10 ng) of bacterial DNA as template, 5 μl of 10× buffer for Taq DNA polymerase (100 mM of Tris–HCl and 15 mM MgCl_2_), 1 μl of MgCl_2_ (25 mM), so that the final concentration of Mg^2+^ is 2 mM, 0.25 μM of each primer, 400 μM of each dNTPs and one unit of Taq DNA polymerase (Bangalore Genei, India). The reaction condition includes an initial denaturation of 7 min at 95 °C, followed by 35 cycles of 1 min at 94 °C, 1 min at 52 °C and 1 min at 72 °C with the final extension of 5 min at 72 °C.

Ps-PCR primer set included forward primer, Ps-for (5′ GGTCTGAGAGGATGATCAGT 3′) and the reverse primer, Ps-rev (5′ TTAGCTCCACCTCGCGGC 3′) for amplification of 990 bp *Pseudomonas*-specific gene region. *rpo*D primer set contains primers PsEG30F 5′ ATYGAAATCGCCAARCG 3′ and PsEG790R 5′ CGGTTGATKTCCTTGA 3′ used for the amplification of 736 bp region. For amplification of both of these primer sets, a 50 μl of reaction mixture included, 5 μl (5–10 ng) of bacterial DNA as template, 5 μl of 10× buffer for Taq DNA polymerase (100 mM of Tris–HCl and 15 mM MgCl_2_), 2.5 μM of each primer, 250 μM of dNTPs and one unit of Taq DNA polymerase (Bangalore Genei, India). The reaction condition for Ps-PCR amplification includes an initial denaturation of 5 min at 95 °C, followed by 35 cycles of 1 min at 94 °C, 1 min at 57 °C and 1 min at 72 °C with the final extension of 10 min at 72 °C. The reaction condition for *rpo*D gene region primer includes an initial denaturation of 7 min at 95 °C, followed by 35 cycles of 5 min at 94 °C, 1 min at 55 °C and 1.5 min at 72 °C with the final extension of 10 min at 72 °C. The amplification was carried out on Gen Amp PCR System 9700 (Applied Biosystems). Amplified DNA was electrophoresed in 1 % agarose gel at 80 mA for 1 h and visualized under UV gel documentation system Gel Doc Mega (BIOSYSTEMATICA).

### Polymorphism in 16S rDNA, *Pseudomonas*-specific and *rpo*D gene region

Restriction digestion of complete 16S rDNA region (1492 bp), *Pseudomonas*-specific gene region (990 bp) and *rpo*D gene region (760 bp) amplicons were set in 25 μl reaction mixture containing 20 μl aliquot of an amplicon of all the PCR reactions from both the primers as template, 1× enzyme buffer and 1 U/rxn of each tetra cutting restriction enzymes *Alu*I, *Rsa*I and *Bam*HI. All the three PCR amplicons were digested with *Alu*I, *Rsa*I and *Bam*HI at 37 °C for 2 h. Enzymes were inactivated by mixing the loading dye and kept at −20 °C. The products were analyzed by agarose gel (2.5 %) electrophoresis carried out at 80 V for 2/3 run of gel.

### Phylogenetic relationships of pseudomonad isolates

DNA bands generated by digestion of amplified 16S rDNA, *Pseudomonas*-specific gene and *rpo*D with the three restriction enzymes *Alu*I, *Rsa*I and *Bam*HI were used to construct a UPGMA dendrogram using NTSYSpc version 2.0 software calculating euclidean similarity coefficient. Bands were scored for their presence (1) and absence (0), ignoring their intensities.

### Identification of fluorescent pseudomonads (FLPs) and sequence analysis

The amplified 16S rRNA gene region, *Pseudomonas*-specific gene region and *rpo*D gene region of the selected antagonistic isolates were amplified with same primers and procedure as mentioned above and sequenced directly on 3730 DNA sequencer by ABI big dye terminator technology (Central Instrumental Facility, Biotech Centre, UDSC, Delhi University, South Campus, New Delhi). The acquired sequences were aligned and their consensus sequences were computed with the DNAMAN analysis system (Lynnon Biosoft, Quebec, Canada). Then these consensus sequences were compared with those extracted from GenBank using BLASTN program and aligned using CLUSTAL W program using MEGA 5 software.

## Results

### Genetic profiling

All the 22 antagonistic FLPs were genetically characterized using three genes; 16S rRNA, *Pseudomonas*-specific and *rpo*D; and phylogeny was determined. All the fluorescent *Pseudomonas* species reported till date are genetically placed into eight groups namely *Pseudomonas* *aeruginosa,* *P.* *fluorescens,* *P.* *chlororaphis,* *P.* *stutzeri,* *P.* *syringae,* *P.* *putida,* *P.* *pertucinogena* and *P.* *putida* (Palleroni et al. 1972; Palleroni 1992).

### Comparing 16S rDNA, *Pseudomonas*-specific and *rpo*D gene as phylogenetic markers

An alternative marker to the 16S rRNA for molecular microbial ecology studies is the single-copy gene encoding the RNA polymerase σ subunit, *rpo*D. This study compares the 16S rRNA, *Pseudomonas*-specific and *rpo*D genes from 22 fluorescent pseudomonads genomes.

**Fig. 1:**
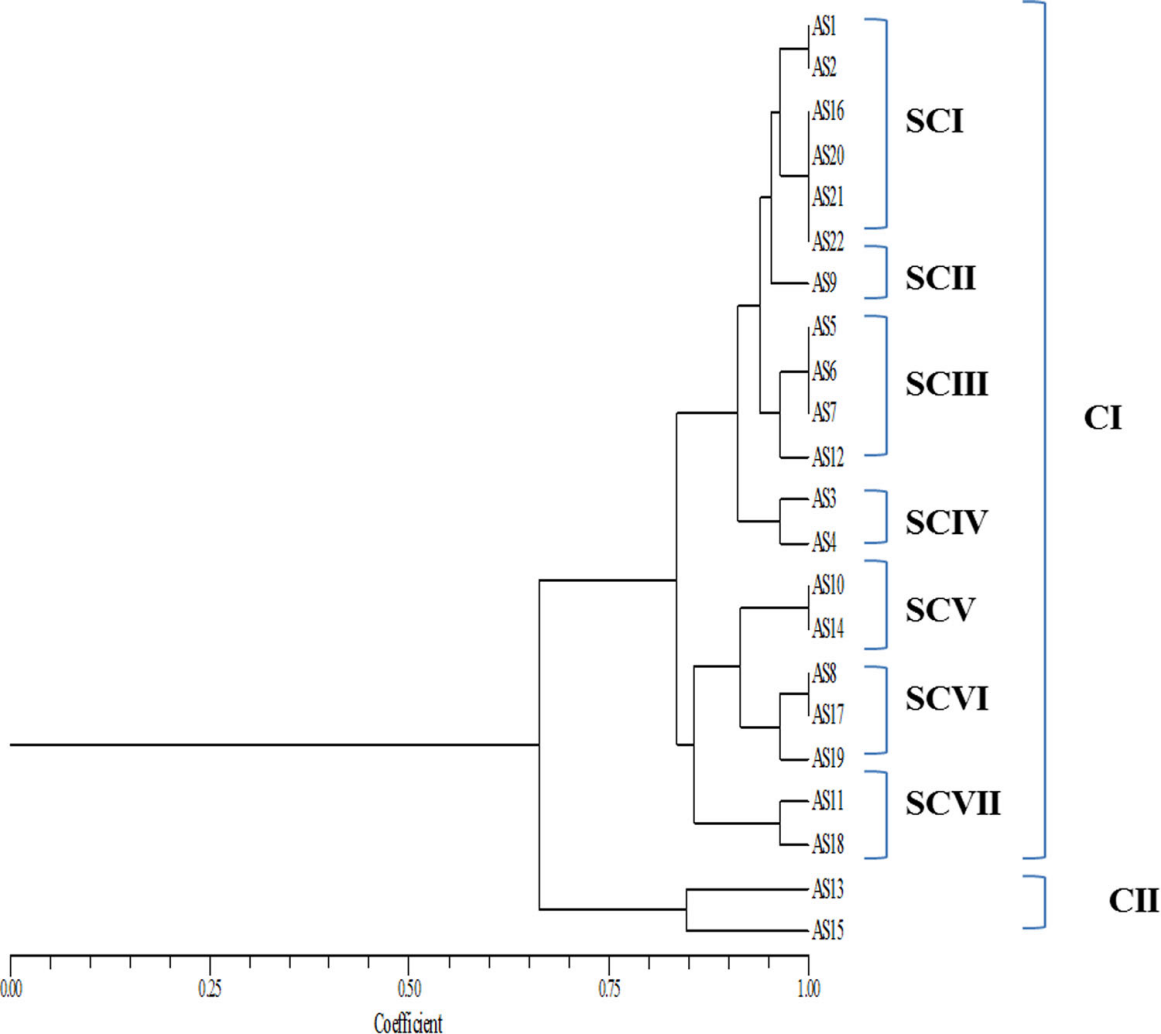
Combined UPGMA dendrogram of 16S rDNA region of FLPs on the basis of ARDRA with *Alu*I, *Rsa*I and *Bam*HI

**Fig. 2:**
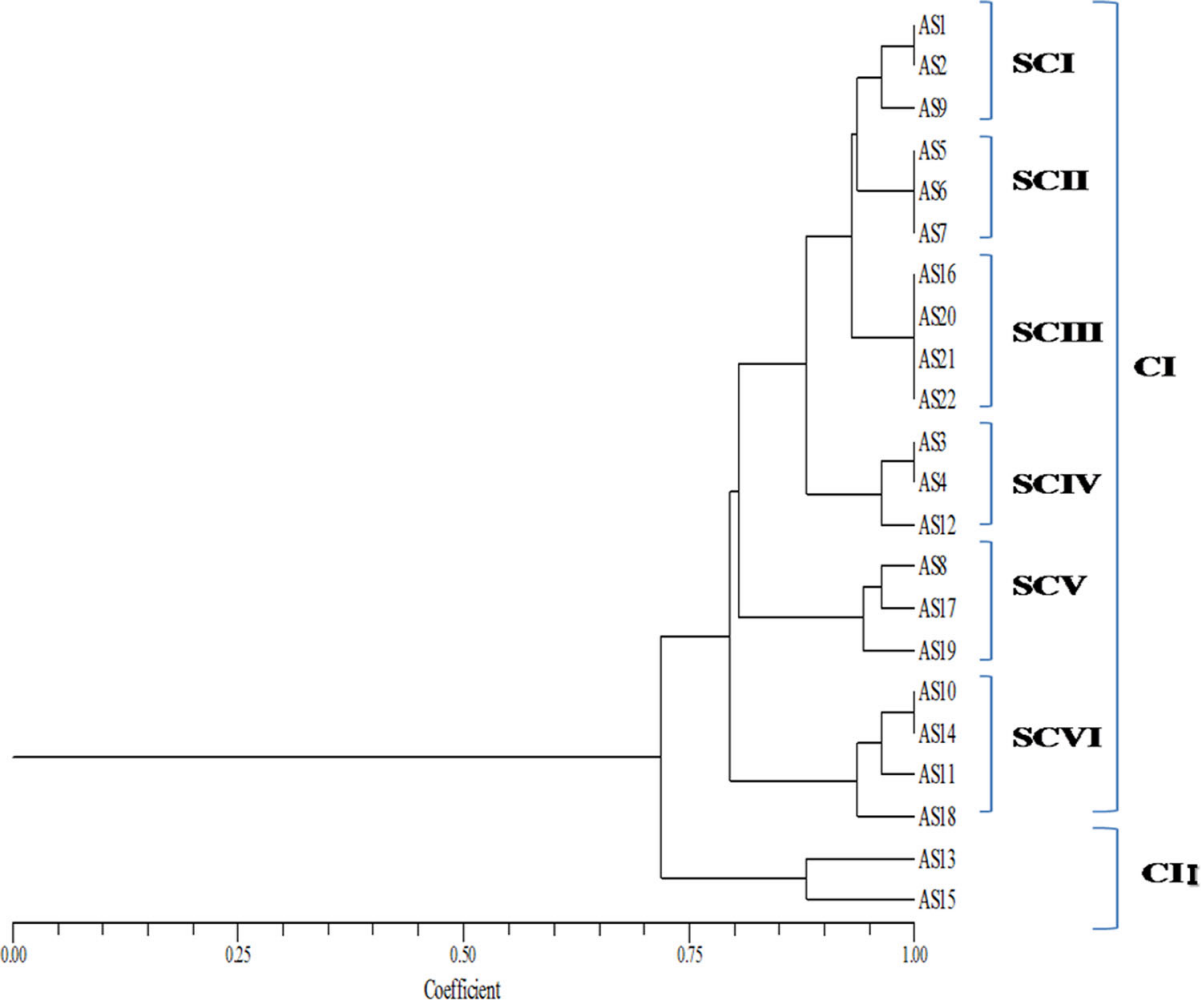
Combined UPGMA dendrogram of *Pseudomonas*-specific gene region of FLPs on the basis of ARDRA with *Alu*I, *Rsa*I and *Bam*HI

The phylogenetic trees obtained from 16SrRNA and *Pseudomonas*-specific genes were almost similar. Cluster I and II delineated in both the dendrogram (Figs. 1, 2), but their relationships within subclusters were modified. Isolate AS9 which was the sole representative of SCII of CI in 16S rDNA-based dendrogram was now placed in SCI of CI along with AS1 and AS2 in dendrogram obtained by *Pseudomonas*-specific gene region. Also isolates AS16, AS20, AS21 and AS22 presented in SCI shifted to SCIII in *Pseudomonas*-specific gene region-based dendrogram. Isolates AS5, AS6 and AS7 presented as SCIII in 16S rDNA-based dendrogram were now placed in a new position as SCII. Similarly, isolates AS8, AS17, AS19 and AS10, AS11, AS14, AS18 were representing two subclusters SC V (AS8, AS17, AS19) and SC VI (AS10, AS11, AS14, AS18) in *Pseudomonas*-specific gene region-based dendrogram while these were arranged within three subclusters in 16S rDNA-based dendrogram SC V (AS10 and AS14), SC VI (AS8, AS17 and AS19) and SC VII (AS11 and AS18).

**Fig. 3:**
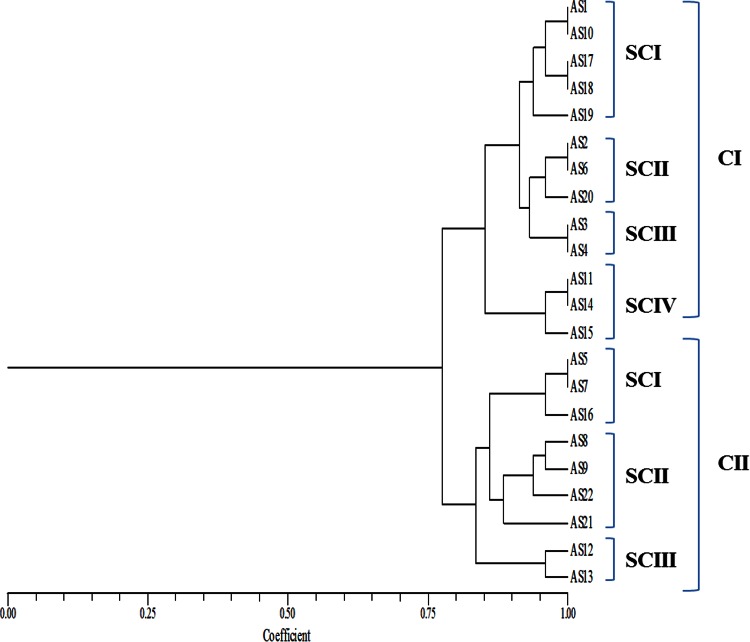
Combined UPGMA dendrogram of *rpo*D gene region of FLPs on the basis of RFLP pattern with *Alu*I, *Rsa*I and *Bam*HI

The relationships of isolates in a dendrogram based on polymorphism in *rpo*D region were different than those obtained with ARDRA and polymorphism in 16S rRNA and *Pseudomonas*-specific region (Fig. 3). In *rpo*D dendrogram, relationships among the FLPs were more refined, there was less euclidean difference among them. All the 22 FLPs were present in two clusters and these two clusters were bifurcated at 0.76 distance on euclidean scale in *rpo*D-based phylogenetic analysis while 16S rDNA-based dendrogram was bifurcated at 0.67 distance and *Pseudomonas*-specific gene region-based dendrogram was bifurcated at 0.71 distance on euclidean scale. From this result it was concluded that *Pseudomonas*-specific gene was more evolved tool for species identification than 16S rDNA while *rpo*D, a housekeeping gene, was best for species identification out of these three chronometers.

### Construction of the phylogenetic structures deduced from nucleotide sequences of the 16S rRNA, *Pseudomonas*-specific and *rpo*D genes

The nucleotide sequences of the 16S rRNA, *Pseudomonas*-specific and *rpo*D genes from three fluorescent pseudomonads were determined, and phylogenetic trees based on these data showing evolutionary relatedness of the isolates along with known *Pseudomonas* species were constructed by the neighbor-joining (NJ) method. Recent approaches used for bacterial characterization include polyphasic system of identification. Three isolates AS5, AS7 and AS15 were selected for further sequencing on the basis of biocontrol traits (data not shown). BLAST analysis of 16S rDNA, *Pseudomonas*-specific gene and *rpo*D gene sequences of biocontrol isolates AS5, AS7 and AS15 showed 99–100 % homology with species of *Pseudomonas*. Gene sequences submitted to GenBank were provided with the accession numbers (Table 1). The sequences of isolates AS5, AS7 and AS15 obtained were compared with those from GenBank using BLASTN (Altschul et al. 1990) and EzTaxon (Kim et al. 2012). For phylogenetic analysis, sequences were aligned using CLUSTAL W software (Thompson et al. 1997). Phylogenetic trees were constructed using neighbor-joining method (Saitou and Nei 1987) by the software MEGA5.

**Table 1:** GenBank accession numbers of the isolates

Code	Name	Accession number
AS5 (16S rDNA region)	*Pseudomonas aeruginosa* strain AS5	KM538944
AS7 (16S rDNA region)	*Pseudomonas aeruginosa* strain AS7	KM538946
AS15 (16S rDNA region)	*Pseudomonas fluorescens* strain AS15	KM538945
AS5 (*Pseudomonas*-specific gene region)	*Pseudomonas aeruginosa* strain AS5	KM538947
AS7 (*Pseudomonas*-specific gene region)	*Pseudomonas aeruginosa* strain AS7	KM538949
AS15 (*Pseudomonas*-specific gene region)	*Pseudomonas fluorescens* strain AS15	KM538948
AS5 (*rpo*D gene region)	*Pseudomonas aeruginosa* strain AS5	KM593260
AS7 (*rpo*D gene region)	*Pseudomonas aeruginosa* strain AS7	KM593262
AS15 (*rpo*D gene region)	*Pseudomonas fluorescens* strain AS15	KM593261

**Fig. 4:**
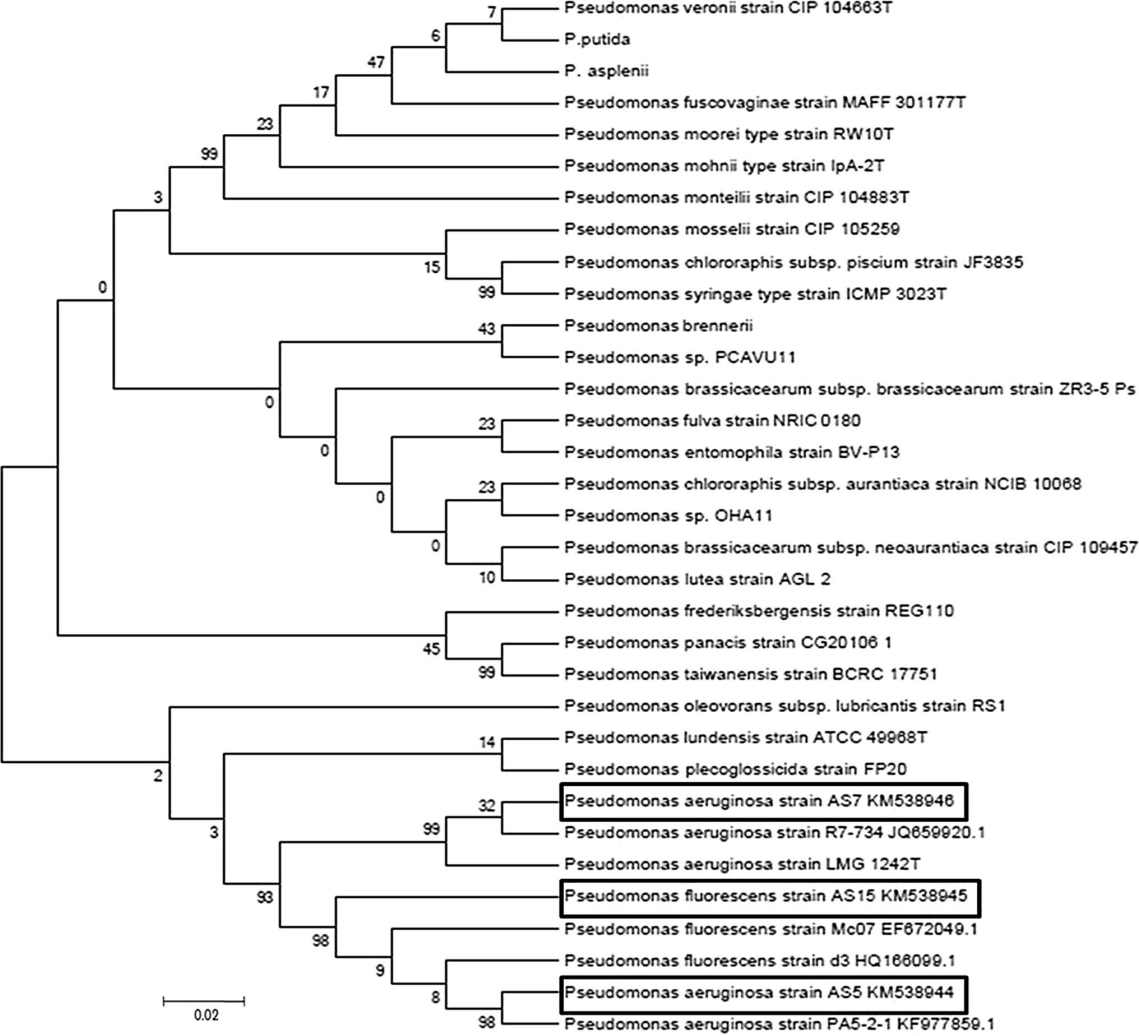
Neighbour-joining phylogenetic tree based on 16S rRNA gene sequences (1492 nt) *Pseudomonas aeruginosa* strain AS5 KM538944, *Pseudomonas aeruginosa* strain AS15 KM538946 and *Pseudomonas fluorescens* strain AS15 KM538945 and the type strains of closely related species of the genus *Pseudomonas*. Bootstrap values (expressed as percentages of 1000 replications) are shown at branch points. *Bar* 2 nt substitutions per 100 nt

The topology of 16S rRNA and *Pseudomonas*-specific gene-based trees was comparable. Comparison of the 16S rRNA gene sequences (Fig. 4) of isolates AS5, AS7 and AS15 against the type strains of *Pseudomonas* species recorded in BLASTN database showed the affiliation of strains to fluorescent groups: *Pseudomonas* *aeruginosa* group and *Pseudomonas* *fluorescens* group, respectively. In this tree, the three fluorescent pseudomonads were grouped into a single cluster, which includes *P.* *plecoglossicida,* *P.* *lundensis* and *P.* *oleovorans* strains along with the *P. aeruginosa* and *P. fluorescens* strains. The closest relatives of *P.* *fluorescens* AS15 were *P.* *aeruginosa*. The next closest relatives are *P.* *plecoglossicida,* *P.* *Lundensis* and *P.* *Oleovorans.* The closest relatives to *P.* *aeruginosa* AS5 and AS7 were *P.* *fluorescens* strains. According to the neighbor joining phylogenetic tree, strains AS5, AS7 and AS15 were clustered in a separate branch most closely related to *P.* *plecoglossicida*. Strains of *P.* *fluorescens* and *P.* *aeruginosa* though present in a separate branch yet not grouped within defined clusters.

**Fig. 5:**
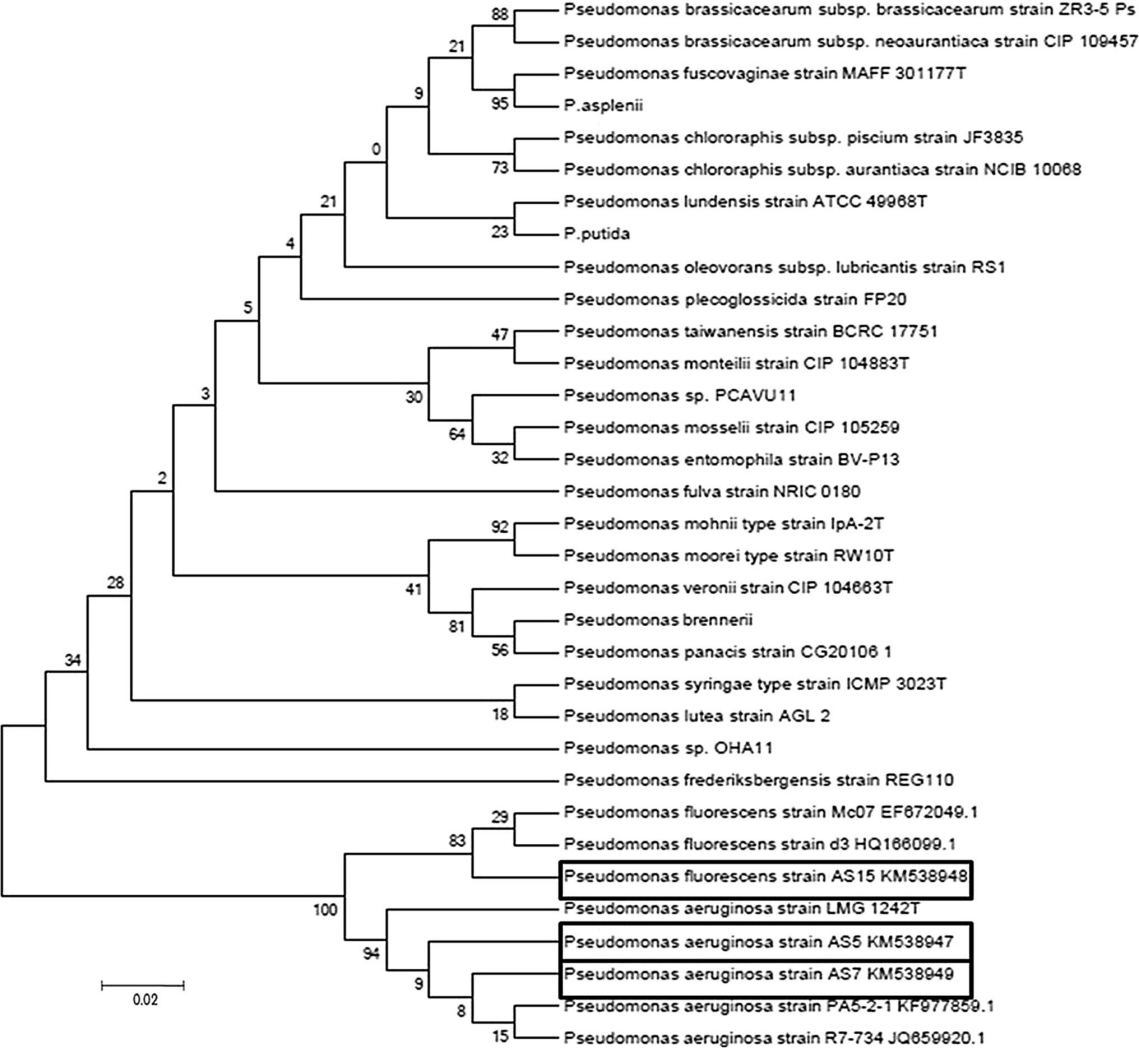
Neighbour-joining phylogenetic tree based on *Pseudomonas*-specific gene sequences (990 nt) *Pseudomonas* *aeruginosa* strain AS5 KM538947, *Pseudomonas aeruginosa* strain AS15 KM538949 and *Pseudomonas fluorescens* strain AS15 KM538948 and the type strains of closely related species of the genus *Pseudomonas*. Bootstrap values (expressed as percentages of 1000 replications) are shown at branch points. *Bar* 2 nt substitutions per 100 nt

Along with 16S rRNA gene, *Pseudomonas*-specific gene was also analyzed (Fig. 5). This gene region was from V2 to V8 variable region of 16S rRNA gene and conserved within genus *Pseudomonas*. As expected the phylogenetic positions of AS5, AS7 and AS15 were similar with that obtained using 16S rRNA gene. In this phylogenetic tree, all the three strains were placed in a separate branch and their closest relative was *P.* *frederiksbergensis*. This concluded that phylogenetic positioning of the isolates within genus *Pseudomonas* with *Pseudomonas*-specific gene and 16S rRNA gene was almost similar. *Pseudomonas*-specific gene defines the phylogenetic position of *P.* *fluorescens* and *P.* *aeruginosa* strains better than 16S rRNA as isolates of both species were grouped into defined clusters.

**Fig. 6:**
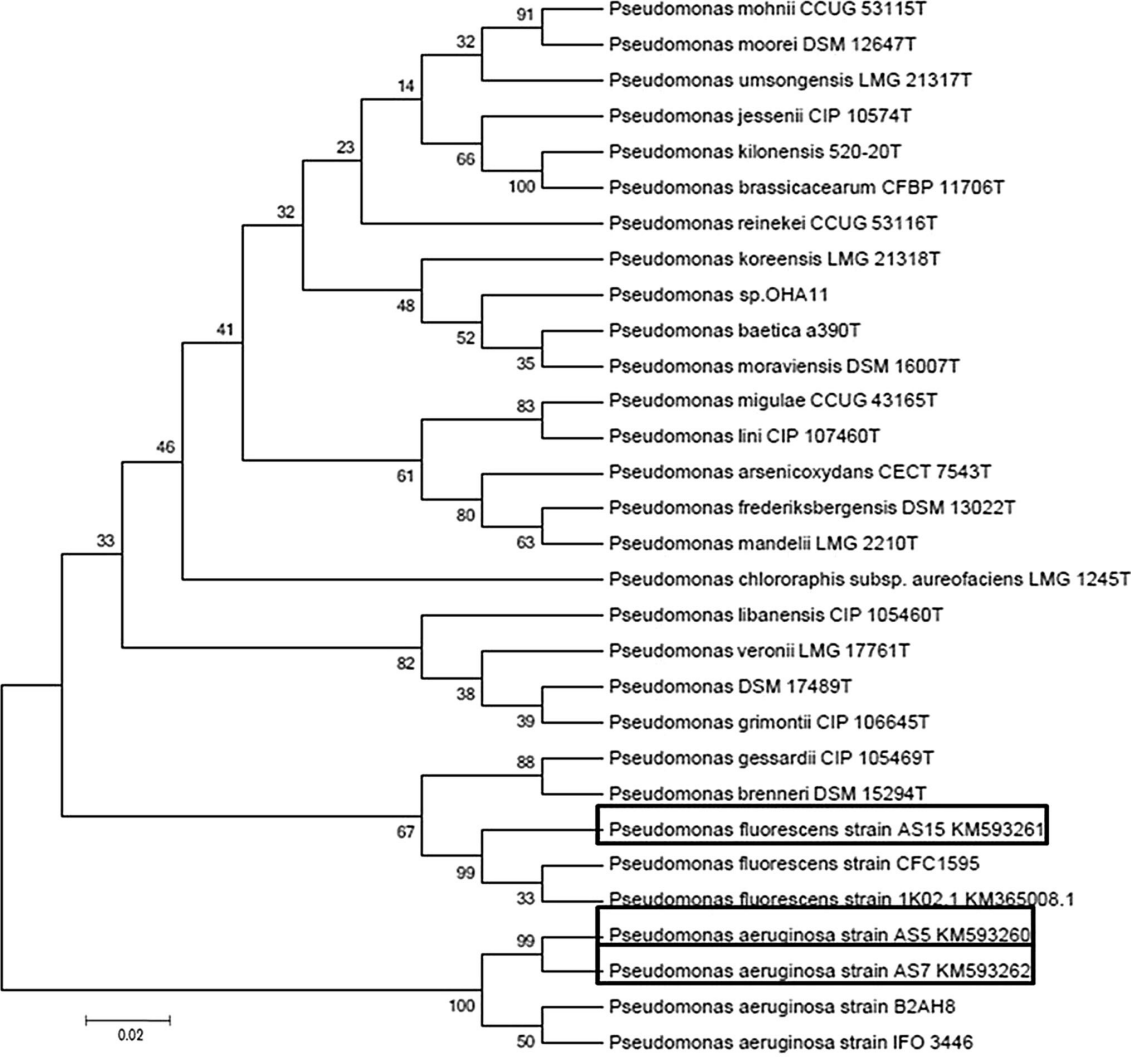
Neighbour-joining phylogenetic tree based on *rpo*D gene sequences (736 nt) *Pseudomonas aeruginosa* strain AS5 KM593260, *Pseudomonas aeruginosa* strain AS15 KM593262 and *Pseudomonas fluorescens* strain AS15 KM593261 and the type strains of closely related species of the genus *Pseudomonas*. Bootstrap values (expressed as percentages of 1000 replications) are shown at branch points. *Bar* 2 nt substitutions per 100 nt

The topology of the tree reconstructed from the *rpo*D gene sequences was different from those obtained from 16S rRNA and *Pseudomonas*-specific gene (Tayeb et al. 2005; Mulet et al. 2009, 2010, 2012; Ramos et al. 2013; Toro et al. 2013). When the whole *rpo*D sequences from the three tested strains were used for the analysis, these were arranged into two major clusters. The *rpo*D gene phylogenetic tree showed that strains AS5 and AS7 (*P.* *aeruginosa*) were clustered in a separate branch from other strains of genus *Pseudomonas*. Strain AS15 (*P.* *fluorescens*) earlier grouped in a same branch with *P.* *aeruginosa* (in 16S rRNA and *Pseudomonas*-specific gene phylogenies), was now placed in a separate branch. Now the closest relatives of AS15 were *P.* *brenneri* and *P.* *gessardii*. In this phylogenetic tree *P.* *fluorescens* and *P.* *aeruginosa* isolates were placed separately into two different branches (Fig. 6).

These topological differences between the 16S rRNA, *Pseudomonas*-specific and *rpo*D-based trees were also observed when the trees were reconstructed by using the unweighted pair group method with averages (UPGMA).

## Discussion

Since all the known FLPs are placed in eight groups (Anzai et al. 2000; Ramette et al. 2011), newly isolated FLPs should be characterized morphologically, biochemically and molecularly to assign it a species epithet. Phylogenetic relationships of pseudomonads resolved by using *rpo*D sequences were eminently different from those resolved by using 16S rRNA and *Pseudomonas*-specific sequences (Moore et al. 1996; Verhille Baïda et al. 1999). These discrepancies appear to have their origins in the eccentric evolutionary process of 16S rRNA genes. The secondary structures of 16S rRNA determined by the complementary sequences in the small helices are functionally important since 16S rRNA provides a scaffold for the assembly of ribosomal proteins into the small subunit and interacts with mRNA. The majority of base substitutions in 16S rRNAs between closely related organisms are located in these helices, called variable regions, and generally these substitutions are compensatory, i.e., they maintain the base pairing within the helices (Rousset et al. 1989; Dixon and Hillis 1993). Yamamoto et al. (2000) observed that the genetic distances in the variable regions of 16S rRNAs correlated poorly with the synonymous distances in the *rpo*D genes. This observation suggests that the base substitutions in these helices might not be accumulated by successive point mutations, but might be caused by single-event mutations introducing multiple substitutions. Thus, the genetic distances calculated from the whole 16S rRNA sequences could be erroneous because the numbers of base substitutions outside the variable regions are much fewer. Because of the high ratios of evolution in the *rpo*D nucleotide sequences, it was very easy to design PCR primers or probes having specificity for clusters of species, subspecies or higher classes. On the other hand, the design of such primers or probes based on the variable regions of 16S rRNA presents a problem, because sequence similarity among the variable regions does not always guarantee a close phylogenetic relationship (Yamamoto et al. 2000). In conclusion, classification, identification and detection systems for pseudomonads based on *rpo*D sequences can be very useful in microbial ecology and other fields of bacteriology.

### Comparing 16S rRNA, *Pseudomonas*-specific and *rpo*D genes as phylogenetic markers

The basic topologies of 16S rRNA and *Pseudomonas*-specific NJ trees were similar to each other, but slightly different from *rpo*D-based NJ tree. The most conspicuous difference between them was the branching order of the clusters that included the ‘*P*. *aeruginosa* lineage’ strains and those that included the ‘*P*. *fluorescens* lineage’ strains. In the 16S rRNA and *Pseudomonas*-specific NJ trees, both of these lineages were branched off as a single cluster after they had diverged from the remaining mass of *Pseudomonas*, whilst in the *rpo*D NJ tree, the ‘*P*. *aeruginosa* lineage’ cluster branched off first, followed by the ‘*P*. *fluorescens* lineage’, which diverged from the rest.

The above results were proved in a study conducted by Case et al. (2006), where phylogenetic resolution by these two genes (16S rRNA and *rpo*D) among 13 data sets showed that the *rpo*D gene provided more phylogenetic resolution than the 16S rRNA gene in seven cases, equal resolution in four cases, and lower resolution in two cases. This concluded that housekeeping genes are better evolutionary chronometer for studying evolutionary relationships among the organisms. Mulet et al. (2009) concluded that the primers designed for *rpo*D gene region are sufficiently selective for detection of *Pseudomonas* because it has only a few degenerations, precisely two for the forward and one for the reverse primer.

### Establishing *rpo*D as an alternative gene marker for microbial ecology

Protein-encoding genes, such as *rpo*D, have several advantages over RNA-encoding genes as molecular markers. They can be used at both the amino acid and nucleotide levels for phylogenetic analysis. Protein alignments allow for resolution of relationships at higher taxonomic levels (domain or phylum) when one or more codon positions are saturated. Nucleotide-level alignments allow for fine-scale resolution, with synonymous first and third codon positions allowing for nearly neutral mutations which can be detected between very closely related organisms (species level or lower). This is likely the reason why *rpo*D performed better than the 16S rRNA gene in resolving relationships at the subspecies level. The resolution provided by *rpo*D can also be increased further by sequencing other protein-encoding genes and examining allelic profiles of different isolates rather than using single gene sequence comparisons (Thompson et al. 1997). Our analyses also demonstrate that *rpo*D displays other important characteristics as an ecological marker, including (a) its universal presence in all prokaryotes; (b) the presence of slowly and quickly evolving regions for the design of probes and primers of differing specificities; (c) having a housekeeping function, making it less susceptible to some forms of lateral gene transfer; and (d) a large enough size to contain phylogenetic information even after removal of regions which are difficult to align. It means that the size of *rpo*D gene is 760 bp which is sufficient to carry the information that will differentiate among the species, i.e., neither the sequence size is too short to carry any useful information nor it too long to have less variable and more conserved sequences which will hinder in differentiation among *Pseudomonas* species.

The use of a single-copy gene for intra-genus species identification is an important milestone in microbial ecology, as it could allow for the accurate measurement of diversity and phylogenetic relationships. *rpo*D provided comparable phylogenetic resolution to that of the 16S rRNA gene at all taxonomic levels, except between closely related organisms (species and subspecies levels), for which it provided better resolution (Case et al. 2006). This is particularly relevant in the context of a growing number of studies focusing on subspecies diversity, in which single-copy protein-encoding genes such as *rpo*B could complement the information provided by the 16S rRNA gene.
